# Supplementary material for: COZOID: contact zone identifier for visual analysis of protein-protein interactions
Source: BMC Bioinformatics. 2018 Apr 6;19:125. doi: 10.1186/s12859-018-2113-6 (PMC5889581; doi:10.1186/s12859-018-2113-6)
Supplement: Supplementary file 2 — Software build. Executable binary file of the software tool. (ZIP 118,784 kb) [file 12859_2018_2113_MOESM2_ESM.zip › cozoid/java/docs/beansbinding-1.2.1-doc.zip!/org/jdesktop/el/package-frame.html]

org.jdesktop.el (Beans Binding)


org.jdesktop.el

Interfaces 
  
*ELContextListener*

Classes 
  
ArrayELResolver
  
BeanELResolver
  
BeanELResolver.BeanProperties
  
BeanELResolver.BeanProperty
  
CompositeELResolver
  
ELContext
  
ELContextEvent
  
ELResolver
  
Expression
  
Expression.ResolvedProperty
  
Expression.Result
  
ExpressionFactory
  
FunctionMapper
  
ListELResolver
  
MapELResolver
  
MethodExpression
  
MethodInfo
  
ResourceBundleELResolver
  
ValueExpression
  
VariableMapper

Enums 
  
Expression.Result.Type

Exceptions 
  
ELException
  
MethodNotFoundException
  
PropertyNotFoundException
  
PropertyNotWritableException
